# Functional Multiplicity of an Insect Cytokine Family Assists Defense Against Environmental Stress

**DOI:** 10.3389/fphys.2019.00222

**Published:** 2019-03-22

**Authors:** Stephen B. Shears, Yoichi Hayakawa

**Affiliations:** ^1^ Inositol Signalling Group, Signal Transduction Laboratory, National Institute of Environmental Health Sciences, National Institutes of Health, Durham, NC, United States; ^2^ Department of Applied Biological Sciences, Saga University, Saga, Japan

**Keywords:** cytokine, growth-blocking peptide (GBP), stress-responsive peptide (SRP), Mthl10, hormesis

## Abstract

The widespread distribution of insects over many ecological niches owes much to evolution of multiple mechanisms to defend against environmental stress, especially because their ectothermic nature and small body size render them particularly susceptible to extremes in temperature and water availability. In this review, we will summarize the latest information describing a single, multifunctional cytokine family that is deployed by six orders of insect species to combat a diverse variety of environmental stresses. The originating member of this peptide family was identified in *Mythimna* (formerly called *Pseudaletia*) *separata* armyworm; the cytokine was named growth-blocking peptide (GBP), reflecting its actions in combating parasitic invasion. The peptide’s name has been retained, though the list of its regulatory activities has greatly expanded. All members of this family are small peptides, 19–25 amino acid residues, whose major source is fat body. They are now known to regulate embryonic morphogenesis, larval growth rates, feeding activities, immune responses, nutrition, and aging. In this review, we will describe recent developments in our understanding of the mechanisms of action of the GBP family, but we will also highlight remaining gaps in our knowledge.

## Introdution

Growth-blocking peptide (GBP) was initially found as a peptidergic factor which blocks JH esterase activation in the hemolymph of early last instar larvae of host *Mythimna* (formerly called *Pseudaletia*) *separata* armyworm upon infection by the parasitic wasp *Cotesia kariyai* (Hayakawa, 1990). GBP-induced suppression of hemolymph JH esterase is a protective measure that delays larval growth and development (Hayakawa, 1991). Although the mechanism by which *M. separata* (*Ms*) GBP suppresses hemolymph JH esterase activation is still unknown, this initial observation led us to focus on its hormone-like function (Hayakawa, 1992). Further characterization of *Ms*GBP signaling elucidated that it elevates dopamine concentrations in the hemolymph through enhanced expression of tyrosine hydroxylase and DOPA decarboxylase in the integument and the brain (Noguchi et al., 1995, 2003). This up-regulation of gene expression was subsequently attributed to *Ms*GBP-induced activation of phospholipase C (PLC), release of inositol triphosphate (IP3), and the elevation of cytoplasmic Ca^2+^ concentrations (Ninomiya and Hayakawa, 2007; Ninomiya et al., 2008). Although the relationship between dopamine elevation and JH esterase repression has not been yet clarified, both events have negative impact on the growth rates of insect larvae (Noguchi et al., 1995).

In the years since the discovery of *Ms*GBP, over 10 GBP orthologous peptides have been found in several lepidopteran species (Hayakawa, 1995, 2006). They all consist of 23–25 amino acids, and they share more than 70% sequence identity, yet they show diverse functions: paralysis induction, plasmatocyte spreading, and cardioacceleration. However, to date, the only known receptor for a GBP is that identified in *Drosophila*-Mthl10 (see below). *Ms*GBP itself was demonstrated to have this multifunctionality (Strand et al., 2000). Subsequent studies established further functions of the GBP family such as cell growth activator, early morphogenetic mediator, and humoral immune mediator (Ohnishi et al., 2001; Tsuzuki et al., 2005, 2012). Nevertheless, as is common practice, this cytokine family is still named after its originating function as a growth-blocking peptide (Hayakawa, 2006).

## Nonlepidopteran GBP

**Figure 1:**
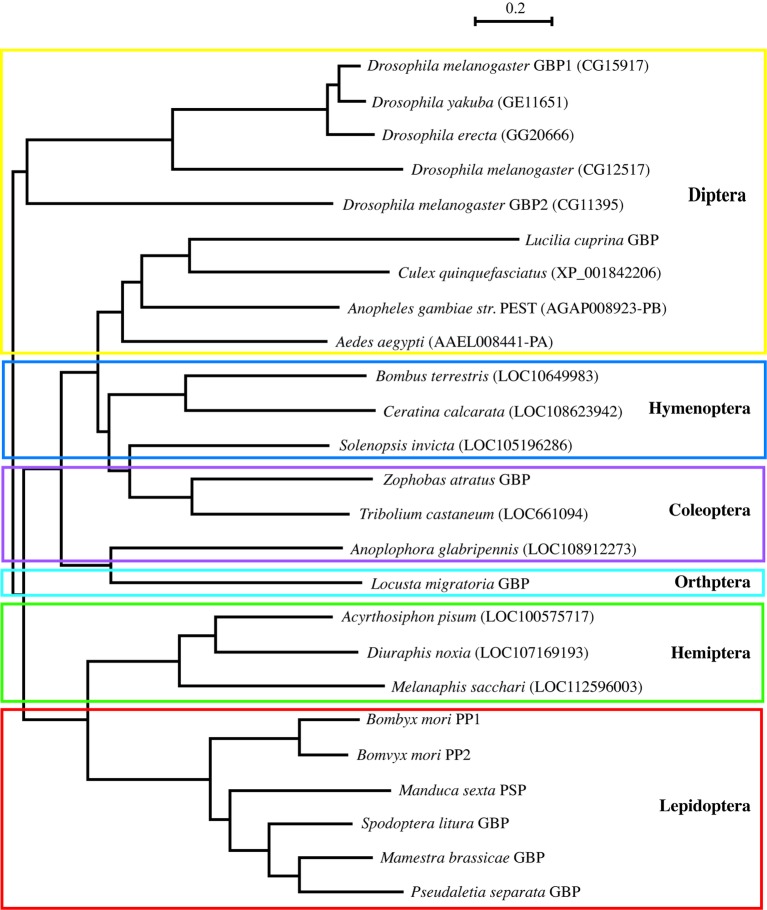
A phylogenetic tree derived from precursor polypeptide sequences of GBP and GBP-like gene family spanning six orders of insects by using the neighbor-joining method with protein-Poisson distances (Saitou and Nei, 1987). The following sequences were identified on database of the NCBI/Blast: *Diuraphis noxia* LOC107169193 (XP_015374346.1), *Melanaphis sacchari* LOC112596003 (XP_025197225.1), *Solenopsis invicta* LOC105196286 (XP_011160410), *Ceratina calcarata* LOC108623942 (XP_017878339), and *Anoplophora glabripennis* LOC108912273 (XP_018572983). *Locusta migratoria* GBP was reported by Durressa et al. (Duressa et al., 2015). Other peptide sequences are in the prior report (Matsumoto et al., 2012). PP: paralytic peptide and PSP: plasmatocyte spreading peptide. Scale bar means a number of amino acid substitution per site.

**Figure 2:**
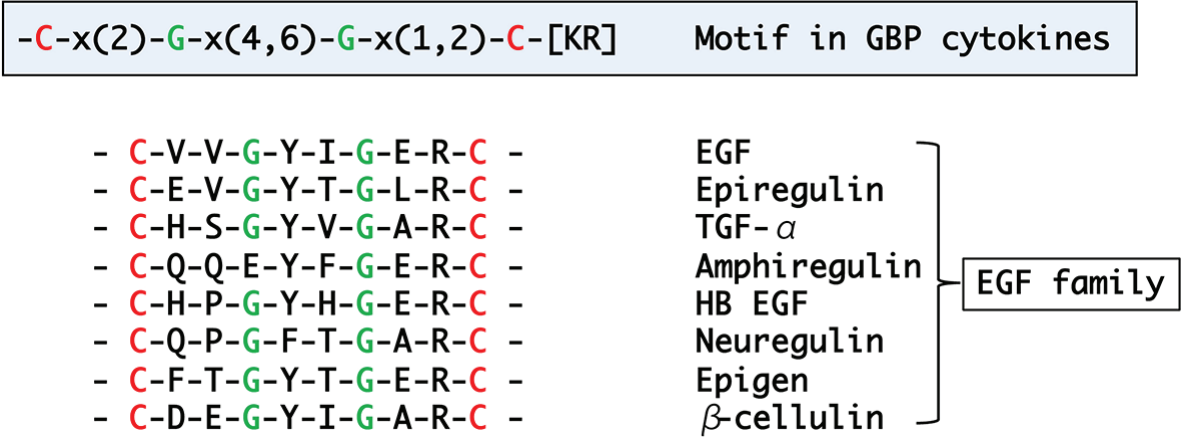
A motif found in the active peptide regions of GBP/GBP-like genes and alignment of mammalian EGF peptide family.

Many GBP orthologous peptides had been reported in Lepidoptera, but it was not until 2012 that the first nonlepidopteran GBP was discovered (Tsuzuki et al., 2012). To identify their primary structures, hemolymph peptides that induce cell growth and plasmatocyte spreading activities were purified from Tenebrionid and bluebottle fly larvae (Matsumoto et al., 2012; Tsuzuki et al., 2012). The functional orthologs identified by these studies comprised 19–24 amino acids, and subsequent homology searches expanded the presence of GBP-like peptides to five orders. Comparisons of these peptides enabled us to extract the consensus motif C-x(2)-G-x(4,6)-G-x(1,2)-C-[KR] (Matsumoto et al., 2012). More recently, this motif has been found in *Locusta migratoria* and *Schistocerca gregaria* GBPs (Duressa et al., 2015). Here, we describe the phylogenetic relationship derived from precursor protein sequences of all known members of the GBP family and GBP orthologs which were identified by homology searches (Figure 1). It is interesting that the GBP motif shares a significant similarity with the portion of the mammalian epidermal growth factor (EGF) motif (Figure 2) that forms the C-terminal region, in which Arg41 and Leu47 have been reported to be crucial for binding to the EGF receptor (Ogiso et al., 2002). NMR analysis demonstrated that the GBP motif core structure (residues 7–22) is predicted to show an EGF-like fold stabilized by a disulfide bond and a short ß-hairpin turn (Aizawa et al., 1999). This characteristic tertiary structure has been reported to be common in lepidopteran GBP orthologs which had been previously referred as to “ENF-peptide” that was named after the consensus N-terminal amino acid sequence (Volkman et al., 1999; Yu et al., 1999).

Although several other insect cytokines, such as Spätzle (DeLotto and DeLotto, 1998; Weber et al., 2003), Unpaired (Zeidler et al., 1999; Karsten et al., 2002; Yang et al., 2015), and Eiger (Moreno et al., 2002), have been reported, most of them were identified by searching for *Drosophila* orthologs of human cytokines. Therefore, GBP is unique in that following its original discovery in the armyworm, and it has since been identified in many other insect species, but no human ortholog has been found yet (Vanha-Aho et al., 2016). It is therefore particularly intriguing that *Drosophila melanogaster* (*Dm*) GBP exhibits some sequence similarity with human defensin BD2, a member of the immunomodulatory ß-defensin family, that can also regulate cell proliferation; BD2 is small, cationic peptides produced by specific proteolytic processing just like *Dm*GBP (Shafee et al., 2017). Furthermore, both the GBP and defensin families recruit the inositol phosphate (IP)/Ca^2+^ signaling cascade to serve their biological actions in common (Niyonsaba et al., 2007; Ninomiya et al., 2008; Zhou et al., 2012; Tsuzuki et al., 2014).

## Drosophila GBP

Following on from the identification of *Dm*GBP, three major developments have been made concerning its functions and signaling mechanisms as follows. First, *Dm*GBP was demonstrated to elevate anti-microbial peptide (AMP) expression independently of the canonical receptors that at that time were known to be associated with the inflammatory pathways mediated by Toll- and IMD-dependent pathways (Tsuzuki et al., 2012). Instead, the adaptor protein IMD is recruited to an activated *Dm*GBP receptor which thereby activates JNK. This signaling pathway stimulates expression of a unique set of AMP genes, mainly *Mechnikowin* and *Diptericin.* The *Dm*GBP-dependent *AMP* expression occurs not only in larvae infected with pathogens but also in larvae exposed to noninfectious stress such as high/low temperatures or mechanical perturbation; thus, GBP has more general roles in maintaining insect homeostasis.

Second, it was demonstrated that *Dm*GBP activates an IP/Ca^2+^ signaling cascade that dictates the timing and the intensity of the separate cellular and humoral components of the innate immune response which, moreover, are reciprocally regulated (Tsuzuki et al., 2014). *Dm*GBP protects against pathogens by activating cellular defense program (phagocytosis and encapsulation), while inhibiting humoral pathways (production and release of AMPs), through an IP/Ca^2+^ signaling-mediated activation of a receptor-regulated kinase cascade (the PVR/ERK pathway).

Third, by screening a dsRNA library that targets genes encoding membrane proteins, the *Dm*GBP receptor has been determined to be the G-protein-coupled receptor *Methuselah-like 10* (*Mthl10*) (Sung et al., 2017). Knockdown of *Mthl10* by RNAi resulted in increased mortality upon bacterial infection and impaired adaptation to an environmental stress such as cold temperature.

It was recently reported that *Dm*GBP regulates the release of insulin-like peptides (ILPs) from the brain depending on nutrient levels in the hemolymph through target of rapamycin (TOR) in *Drosophila* (Koyama and Mirth, 2016). Thus, it was investigated if the GBP elicited those effects by acting through Mthl10. *Mthl10* was found to be expressed in ILP-producing cells of the brain and *Mthl10* knockdown decreased ILP expression (Sung et al., 2017). *Mthl10* knockdown was also demonstrated to be associated with increased longevity of flies, while *DmGBP* overexpression shortened lifespans. Furthermore, the GBP-induced shorter-lived phenotype was not observed in a strain with simultaneous knockdown of *Mthl10.* These observations provided solid evidence that Mthl10-mediated integration of various immunological and metabolic properties of *Dm*GBP is essential to maintain health and homeostasis that are critical for normal lifespan in insects.

## GBP Signaling and its Regulation

In mammals, the cytokine TNF triggers the production of proinflammatory cytokines such as IL-1ß and IL-6 (Cunha et al., 1991; Lorenzetti et al., 2002). Furthermore, IL-6 expression has been demonstrated to be induced by IL-1ß in epithelial cells (Moon et al., 2000; Khan et al., 2014). Another insect cytokine, stress-responsive peptide (SRP), was recently identified; its expression is enhanced by *Ms*GBP in the armyworm (Yamaguchi et al., 2012; Matsumura et al., 2018). Physiological functions of SRP are similar to those of *Ms*GBP. For example, both *Ms*GBP and SRP showed larval growth retardation when they are injected into early last instar larvae. Although *Ms*GBP elicits a slightly stronger growth inhibitory effect than SRP, co-injection of both peptides has a greater effect than that due to *Ms*GBP alone (Yamaguchi et al., 2012; Matsumura et al., 2018). The negative impact on larval growth seems to be mainly due to the *Ms*GBP and/or SRP-induced decrease in larval feeding activities: co-injection of both cytokines caused a slightly more severe reduction in appetite than injection of each individual factor alone. Similar effects by cytokines have been reported in mouse IL-1ß and IL-6: both cytokines synergistically enhanced STAT3/NF-κB-dependent gene expression in the mouse liver during the acute inflammation phase (Goldstein et al., 2017). It might be worth investigating the functional parallelism between *Ms*GBP—SRP and IL-1ß—IL-6 to clarify evolutional feature of cytokine functions. Furthermore, it was demonstrated that *Ms*GBP does not elevate *SRP* expression when injected with SRP into the armyworm larvae (Matsumura et al., 2018), indicating that *Ms*GBP cannot activate *SRP* expression as long as SRP is present in the hemolymph above a threshold concentration. This might be analogous to the fact that an excessive immune response, through strong stress, stimulates a negative feedback mechanism in mammals, which protects the organism from an overproduction of proinflammatory cytokines (Elenkov and Chrousos, 2002).

Another mode of GBP signaling regulation is the control of its hemolymph concentrations by GBP-binding protein (GBP-BP) that functions as a scavenger of *Ms*GBP in the armyworm (Matsumoto et al., 2003). As mentioned above, *Dm*GBP initially tends to prioritize neutralization of an invading pathogen by activating cellular defense reactions (spreading, phagocytosis, and encapsulation). *Ms*GBP regulates not only immune active plasmatocytes and granulocytes in Lepidoptera (Lavine and Strand, 2002), but also another hemocyte class, the oenocytoids. The latter cells possess densely packed GBP-BP molecules, which are released by *Ms*GBP-induced cell lysis that occurs after the cellular immune responses of plasmatocytes (Matsumoto et al., 2003). Therefore, *Ms*GBP has temporally dependent actions, first to stimulate the immune cells and afterwards to silence its own action by releasing GBP-BP through specific hemolysis of oenocytoids. Although an equivalent GBP-BP has not been identified in *Drosophila,* orthologous genes and proteins have been identified in several Lepidoptera such as *Manduca sexta* (Chevignon et al., 2015), *Bombyx mori* (Hu et al., 2006; Sasibhushan et al., 2013), *Spodoptera exigua* (Park and Kim, 2012), *Spodoptera frugiperda* (Barat-Houari et al., 2006), and *Helicoverpa armigera* (Shelby and Popham, 2009). Bacterial and viral infection has been reported to enhance expression of GBP-BP genes in the hemocytes of some lepidopteran larvae, which supports the proposed immunological functional role of this protein. Moreover, expression of GBP-BP is dependent on the dependent stage of the insect and is enhanced by 20-hydroxyecdysone (20E), which together suggests that there are other consequences for the interaction of GBP with GBP-BP (Zhuo et al., 2018). For example, GBP and its binding protein may exert metabolic regulation during metamorphosis; down-regulation of metabolic levels by clearance of hemolymph GBP by GBP-BP would help the normal process of metamorphosis because it is well known that insects become inactive during metamorphosis. Furthermore, it has been shown that there are sharp GBP peaks in the hemolymph during each larval molt (Ohnishi et al., 1995). It is possible that GBP-BP contributes toward purging hemolymph GBP after the shut-off of its gene expression, which could make the sharp GBP peaks during molt periods.

## Future Investigations of GBP Signaling

There remain many important questions regarding GBP multifunctionality and their regulation. For example, it has been demonstrated that GBP serves its immunological and metabolic functions as described above. Furthermore, GBP functions as a cell growth factor (Hayakawa and Ohnishi, 1998; Matsumoto et al., 2012). It has been reported that *Ms*GBP acts as a bipolar growth regulator: high concentrations (over several 10 pmol/ml) suppress larval growth but low concentrations (several pmol/ml) enhance larval growth and cell proliferation (Hayakawa and Ohnishi, 1998). In fact, several pmol/ml of *Ms*GBP enhances proliferation of human keratinocytes and of SF-9 insect cells in a manner similar to mammalian EGF (Hayakawa and Ohnishi, 1998). It is not yet known if Mthl10 contributes to *Dm*GBP-dependent cell proliferation. Indeed, based on the prior results obtained by structural (Aizawa et al., 1999) and kinetic studies (Hayakawa and Ohnishi, 1998; Ohnishi et al., 2001), it is reasonable to expect that stimulation of cell growth by GBP requires another type of the receptor similar to the EGF family of receptor tyrosine kinases. The speculation that GBP could activate multiple receptor types has arisen from the demonstration that different minimal peptide sequences of *Ms*GBP are required for cell growth and cellular immune activities: residues 2–23 in GBP are required for the former activity and 1–22 in GBP for the latter (Aizawa et al., 2001).

When *Dm*GBP (CG15917) was first identified, four other *Drosophila* genes encoding the proGBP-like peptide were also found: CG11395, CG12517, CG14069, and CG17244. Koyama and Mirth recently found that the CG11397 gene product regulated the release of ILPs from the brain in the similar manner of GBP (CG15917) and they named CG15917 and CG11397 for GBP1 and GBP2, respectively (Koyama and Mirth, 2016). The role of these two *Dm*GBPs in metabolic regulation has been demonstrated, but it has not yet been checked whether GBP2 also shares similar immune regulatory functions with GBP1. Moreover, it remains to be seen if the other candidate genes described above (CG12517, CG14069, and CG17244) will turn out to expand the functionality of the *Dm*GBP family.

## Conclusions

Although the multiple functionalities of *Ms*GBP and *Dm*GBP have been clearly demonstrated, it remains unclear to what extent the GBP-signaling pathways and functionalities are conserved in other insects. For example, it is not yet known if *Mthl10* orthologous gene occurs in the armyworm. Moreover, SRP and GBP-BP have been examined only in the armyworm. It will be important to identify all these essential components for GBP-signaling function and regulation in broad insect species, so as to identify species-, development-, and stage-specific expression of such components. Elucidating commonality and difference of such GBP-associated factors in insects may hint at the conservation of some of these important homeostatic mechanisms in mammals.

## Author Contributions

All authors listed have made a substantial, direct and intellectual contribution to the work, and approved it for publication.

### Conflict of Interest Statement

The authors declare that the research was conducted in the absence of any commercial or financial relationships that could be construed as a potential conflict of interest.
